# Long-term moderate calorie restriction inhibits inflammation without impairing cell-mediated immunity: a randomized controlled trial in non-obese humans

**DOI:** 10.18632/aging.100994

**Published:** 2016-07-13

**Authors:** Simin N. Meydani, Sai K. Das, Carl F. Pieper, Michael R. Lewis, Sam Klein, Vishwa D. Dixit, Alok K. Gupta, Dennis T. Villareal, Manjushri Bhapkar, Megan Huang, Paul J. Fuss, Susan B. Roberts, John O. Holloszy, Luigi Fontana

**Affiliations:** ^1^ The Jean Mayer USDA Human Nutrition Research Center on Aging at Tufts University, Boston, MA 02111, USA; ^2^ Duke University Medical Center, Durham, NC 27705, USA; ^3^ University of Vermont, Burlington, VT 05405, USA; ^4^ Department of Medicine, Washington University School of Medicine, St Louis, MO 63110, USA; ^5^ Comparative Medicine and Department of Immunobiology, Yale University School of Medicine, New Haven, CT 06510, USA; ^6^ Pennington Biomedical Research Center, Baton Rouge, LA 70808, USA; ^7^ Baylor College of Medicine and Michael E DeBakey VA Medical Center, Houston, TX 77030, USA; ^8^ Department of Clinical and Experimental Sciences, Brescia University School of Medicine, Brescia, Italy; ^9^ CEINGE Biotecnologie Avanzate, Napoli, Italy

**Keywords:** human, familial longevity, calorie restriction, inflammation, vaccine response, cell-mediated immunity

## Abstract

Calorie restriction (CR) inhibits inflammation and slows aging in many animal species, but in rodents housed in pathogen-free facilities, CR impairs immunity against certain pathogens. However, little is known about the effects of long-term moderate CR on immune function in humans. In this multi-center, randomized clinical trial to determine CR's effect on inflammation and cell-mediated immunity, 218 healthy non-obese adults (20-50 y), were assigned 25% CR (n=143) or an ad-libitum (AL) diet (n=75), and outcomes tested at baseline, 12, and 24 months of CR. CR induced a 10.4% weight loss over the 2-y period. Relative to AL group, CR reduced circulating inflammatory markers, including total WBC and lymphocyte counts, ICAM-1 and leptin. Serum CRP and TNF-α concentrations were about 40% and 50% lower in CR group, respectively. CR had no effect on the delayed-type hypersensitivity skin response or antibody response to vaccines, nor did it cause difference in clinically significant infections. In conclusion, long-term moderate CR without malnutrition induces a significant and persistent inhibition of inflammation without impairing key *in vivo* indicators of cell-mediated immunity. Given the established role of these pro-inflammatory molecules in the pathogenesis of multiple chronic diseases, these CR-induced adaptations suggest a shift toward a healthy phenotype.

## INTRODUCTION

Calorie restriction (CR) without malnutrition is the most powerful intervention to increase lifespan in simple model organisms and rodents [1]. CR decreases inflammation, which is believed to protect against age-associated diseases [2, 3]. Low-grade chronic inflammation is deeply implicated in the pathogenesis of multiple age-associated chronic diseases and in the biology of aging itself [4]. Serum concentrations of C-reactive protein (CRP, a highly specific systemic marker of inflammation) and TNF-α (a powerful pro-inflammatory cytokine) are both associated with an increased risk of developing insulin resistance, type 2 diabetes (T2D), cardiovascular disease (CVD) and cancer [5-8]. Excessive adiposity is associated with increased adipose tissue TNF-α expression [9] and serum TNF-α levels [10], which are reduced by weight loss [9, 11]. However, concerns exist regarding the potential immunosuppressive effects of CR, because some studies have shown a detrimental effect on cell-mediated immune responses in monkeys [12] and increased susceptibility to infection in rodents [13, 14]. On the contrary, other studies in aging mouse and monkeys show that CR can enhance the T cell receptor diversity suggesting improved immune –surveillance [15, 16].

In humans, CR including a restriction of protein and essential nutrients impairs cell-mediated immune responses [17] and increases susceptibility to morbidity and mortality from infectious diseases. However, little is known about the long-term effects of moderate CR with adequate intake of nutrients on inflammatory markers and cell-mediated immune response of healthy adults.

A purpose of this 2-year multicenter randomized controlled trial (RCT) was to evaluate the effects of a 25% CR diet on inflammatory markers [WBC count, high sensitivity CRP (hs-CRP), pro-inflammatory cytokines, adhesion molecules], and *in vivo* measures of cell-mediated immunity [antibody response to 3 vaccines, and delayed-type hypersensitivity skin response (DTH) to three recall antigens] in a large number of healthy, non-obese young and middle-aged individuals. Self-reported infections, allergies and related medications were documented.

## RESULTS

### Participants and baseline characteristics

**Figure 1:**
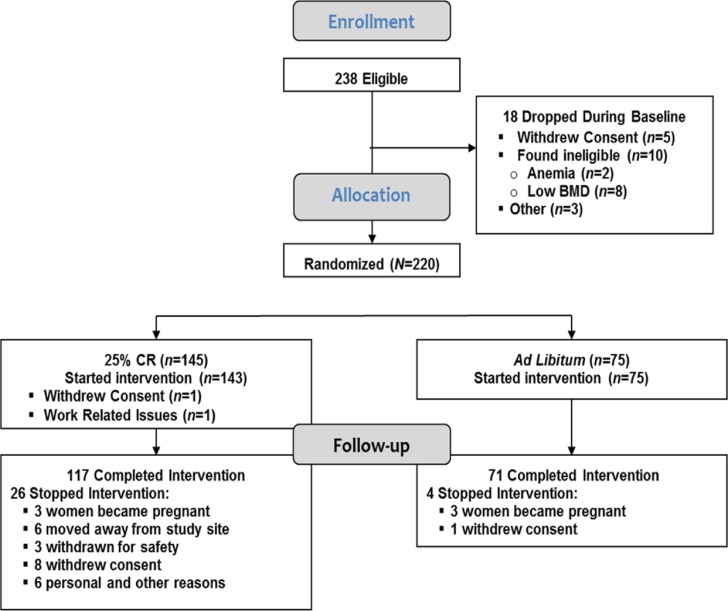
CONSORT diagram Two hundred and thirty eight individuals were eligible and 220 individuals were randomized. Two individuals, both assigned to the calorie-restricted (CR) group, dropped out prior to starting the intervention, resulting in an intention-to-treat cohort of 218 participants; 75 in the ad libitum (AL) control and 143 in the CR group (Table 1). Thirty participants were withdrawn or dropped from the intervention prior to completion including 4 (5.3%) in the AL control group and 26 (18.2%) in the CR group (p=0.01).

As described previously [18], 1,069 interested individuals were invited to an in-person screening evaluation, 238 started baseline testing and 220 were randomized. Two CR participants dropped prior to randomization, resulting in an ITT cohort of 218 (Figure 1 and Table 1). Thirty participants withdrew from the study [4 (5.3%) in the AL and 26 (18.2%) in the CR group (p= 0.01)]. Three CR participants continued the study evaluations beyond withdrawal and were included in analyses. There were no differences at baseline between AL and CR groups in biometric and demographic variables including body weight, body mass index (BMI) and other body composition and demographic variables, blood glucose or lipid profile (Table 1) or for any of the immune and inflammatory outcomes.

**Table 1:** Demographic, anthropometric and clinical characteristics at baseline for the 218 participants who started the 2-year intervention *

	Calorie Restriction (n=143)^†^	Ad Libitum (n=75)^†^
Race		
White, n (%)	111 (77.6%)	57 (76%)
African American, n (%)	15 (10.5%)	11 (14.7%)
Other, n (%)	17 (11.9%)	7 (9.3%)
Sex (F/M)	99F/44M	53F/22M
Age, y	38.0 (7.2)	37.9 (6.9)
Height, cm	168.9 (8.6)	168.4 (8.3)
Baseline Weight, kg	71.8 (9.2)	71.3 (8.6)
Baseline BMI, kg/m^2^	25.1 (1.7)	25.1 (1.6)
Body Fat, %	33.6 (6.6)	32.9 (6.1)
*Blood pressure*		
SBP, mmHg	112 (9.9)	111 (9.9)
DBP, mmHg	72.1 (7.5)	71.2 (7.1)
*Laboratory Values*		
Glucose, mg/dL	81.9 (5.6)	83.6 (6.1)
Insulin, μIU/mL	5.4 (0.2)	5.8 (0.3)
HDL-C, mg/dL	49.1 (13.3)	49.2 (11.7)
LDL-C, mg/dL	98.0 (26.5)	105.6 (28.6)
Tg, mg/dL	103.5 (50.5)	106.8 (59.7)

Abbreviations: AL, ad libitum control group; CR, 25% calorie restriction group; SBP, systolic blood pressure; DBP, diastolic blood pressure; HDL-C, high density lipoprotein cholesterol; LDL-C, low density lipoprotein cholesterol; Tg, triglycerides.

* Values represent mean (SD).

† No significant between group differences for all listed variables.

### Intervention adherence and body composition

Participant adherence and changes in body composition in response to CR have been published elsewhere [19]. Energy intake was reduced by 19.5 (0.8) % (480 kcal/d) during the first 6-months of CR, and by an average of 9.1 (0.7) % (234 kcal/d) for the remaining 18-mo (p<0.0001 vs. AL). CR induced significant reduction in body weight [8.3 (0.3) kg (11.5%) at 1-y and a net change of 7.6 (0.3) kg (10.4%) at 2-y (p<0.001)], BMI and % body fat [19]. No significant change was observed in energy intake or body composition in the AL group. Measured by DEXA, CR induced a 6.1% (0.2) kg change in Fat Mass at 1-yr and 5.3 (0.3) kg at 2-yr, but did not change in the AL group.

### Moderate CR impacts white blood cell profile

**Figure 2:**
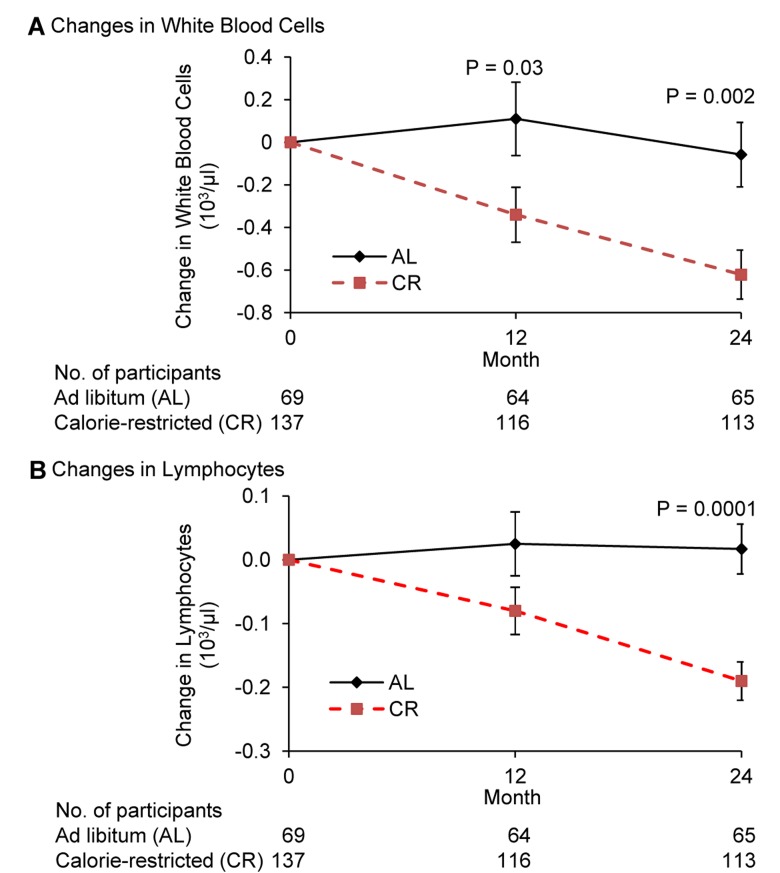
Change in the number of white blood cells and lymphocytes following 2 years of calorie restriction in humans Panel (**A**) baseline values of white blood cells for ad libitum (AL) and calorie-restricted (CR) groups were 5.9 × 10^3^/μl and 6.0 × 10^3^/μl, respectively. Panel (**B**) baseline values of lymphocytes for both AL and CR groups were 1.8 × 10^3^/μl. Data are mean (SE). The P value comparisons are for AL and CR groups at indicated time points.

Complete blood count and differentials (CBC-Diff) stayed within normal ranges in both groups. However, compared to AL, CR significantly reduced the number of WBC at month 12 (p=0.03), and 24 (p=0.002) (Figure 2A). There was a trend for a correlation between changes in BMI from baseline to 24 months and that of WBC number (r=0.14, p=0.07) when both CR and AL groups were combined.

Compared to AL, CR significantly reduced the number of lymphocytes at month 24 (p=0.0001) (Figure 2B). The difference in the change in lymphocytes between CR and AL group was −0.106 at 12 months (p=0.09) and −0.207 at month 24. (p<0.0001. A significant correlation between changes in BMI from baseline to month 24 and that of lymphocytes (r=0.20, p=0.006) was observed when both CR and AL groups were combined.

While a significant difference in change in monocytes was observed between the two groups, this was mainly due to an increase in the AL group. The decrease in neutrophils in the CR group at month 24 in comparison to the AL group tended to be significant (p=0.067) (Supplemental Table 1). No significant differences in the eosinophils or basophils were observed (Both groups showed a small but significant increase in basophils; these numbers stayed within normal ranges (Supplemental Table 1).

### Moderate CR reduces circulating inflammatory markers

**Figure 3:**
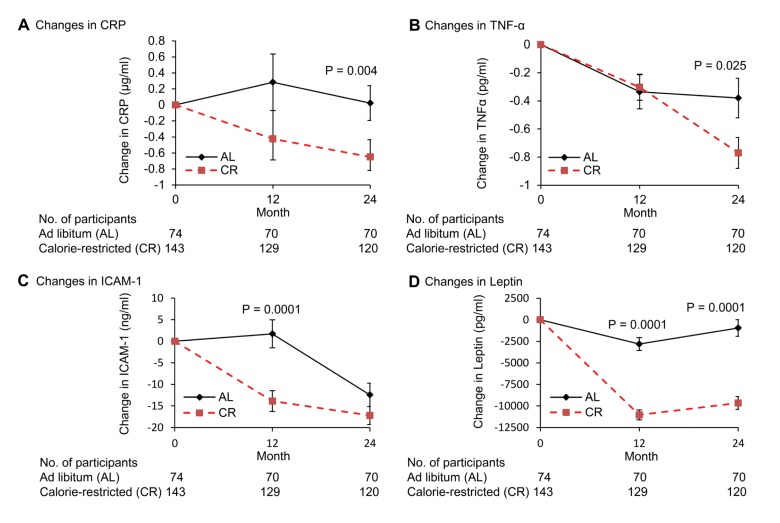
Change in plasma concentrations of inflammation markers following 2 years of calorie restriction in humans Panel (**A**) baseline values of C-reactive protein (hs-CRP) for ad libitum (AL) and calorie-restricted (CR) groups were 1.1 and 1.5 μg/mL, respectively. Panel (**B**) baseline values of tumor necrosis factor-alpha (TNF-α) for AL and CR groups were 3.1 and 3.5 pg/ml, respectively. Panel (**C**) baseline values of intercellular adhesion molecule-1 (ICAM-1) for AL and CR groups were 165.4 and 165.0 ng/ml, respectively. Panel (**D**) baseline values of leptin for AL and CR groups were 17.7 and 16.9 ng/ml, respectively. Data are mean (SE). The P value comparisons are for AL and CR groups at indicated time points.

CRP (natLog) decreased significantly in the CR compared to AL group at both months 12 and 24 (p=0.001) (Figure 3A). The correlation between change in BMI and change in the natural logarithm of CRP trended toward significance (r=0.15, p=0.05).

Plasma TNF-α decreased significantly in both AL and CR groups at month 12 (−0.34 vs. −0.30 pg/mL; p=0.012, p=0.0024 for AL and CR, respectively); further declines in the CR group between month 12 and 24 (p=0.018) resulted in a significantly higher decrease in TNF-α in CR compared to AL group at month 24 (p=0.025) (Figure 3B). A significant correlation between changes in BMI from baseline to 24 months and that of TNFα (r=0.15, p=0.04) was observed when both CR and AL groups were combined.

Compared to AL group, there was a significant decline in serum ICAM-1 levels in the CR group from baseline to month 12 (P<0.0001), however, ICAM levels in the AL group decreased significantly between month 12 and 24 (P<0.0001) resulting in a non-significant difference between the AL and CR groups at month 24 (P=0.14) (Figure 3C). A significant correlation between change in BMI from baseline to 24 months and that of ICAM-1 (r=0.17, p=0.02) was observed when both CR and AL groups were combined.

The change in leptin level was significantly greater in CR compared to AL group at both month 12 and 24 (p<0.0001) (Figure 3D). In addition, a significant correlation was observed between changes in BMI from baseline to month 24 months and changes in leptin when both CR and AL groups were combined (r=0.60, p=0.001).

No significant changes were observed for IL-6, IL-8, and MCP-1 (data not shown).

### Response to vaccine

Antibody responses to vaccines were measured at the end of the intervention. Three vaccines, Hepatitis A (HEP-A) (primary T cell-dependent), tetanus/diphtheria (TD) (secondary T cell-dependent) and pneumococcal (B cell dependent) (PN) were administered at month 17. A booster shot for HEP-A was administered at month 23. Blood for antibody response was collected at month 17 (before vaccination), 18, and 24 (after vaccination) for all vaccines, and 23 for before HEP-A booster. There was no significant difference between AL and CR groups in pre-vaccination (month 17 and 23) or post-vaccination (month 18 and 24) levels of antibodies to Hepatits A HEP-A, TD), or any of the PN IgG serotypes (1, 2, 3, 4, 5, 6, 7F, 8, 9N, 9V, 10A, 11A, 12F, 14, 15B, 17F, 18C, 19A, 19F, 20, 22F, 23F, 33F) (Supplemental Table 3).

For HEP-A antibody level, the majority of subjects within both AL and CR groups had levels above the detection limit and thus quantitative values could not be obtained. However, there were no differences between AL and CR groups in % participants who had values above the detection limit at any time point for HEP-A or other vaccines.

### DTH

There was no significant difference at baseline between AL and CR groups in the diameter of induration at 24 or 48 h for individual antigens or for total diameter of induration (Table 2), nor in number of positive antigens. There were no significant main effects of treatment or time and their interaction or in the change during 2-years in the total number of positive antigens or total diameter of induration for positive responses (≥5mm) or all observed responses at 24 or 48 hours between CR and AL groups. A significant within CR group change from baseline to month 24 (p=0.001) in total diameter of induration (Table 2) was observed and both groups showed a significant decline in the diameter of induration for positive responses (values ≥5mm) to *Tetanus toxoid* (p=0.016). The reason for this decline is not clear and cannot be explained by any methodological inconsistency, changes in participants' health status, timing of administration of DTH, or timing of tetanus vaccination.

**Table 2:** Effect of calorie restriction on delayed-type hypersensitivity skin response at 48 hours*

	Time point		
Variable	Baseline	Month 12	Month 24
Trichophyton (diameter of induration, mm)			
AL	0.9 (0.3)	2.2 (0.6)	3.1 (0.8)
CR	1.5 (0.4)	1.1 (0.5)	1.8 (0.7)
p-value	0.79	0.294	0.381
Tetanus (diameter of induration, mm)			
AL	12.1 (1.1)	10.5 (1.3)	9.7 (1.1)†, ‡
CR	13.5 (1.0)	10.5 (1.0)†	8.0 (0.9)†
p-value	0.812	1	0.407
Candida (diameter of induration, mm)			
AL	7.5 (0.9)	9.2 (1.3)	9.4 (1.2)
CR	9.5 (0.8)	10.0 (1.0)	8.7 (0.9)
p-value	0.167	1	1
Total Diameter of Induration (mm) for all observed values			
AL	20.5 (1.7)	20.6 (2.2)	21.5 (1.9)
CR	24.4 (1.4)	21.2 (1.6)	18.2 (1.6)†, §
p-value	0.127	1	0.353
Number of Positive Responses (≥ 5mm)			
AL	1.48 (0.09)	1.64 (0.11)	1.72 (0.13)
CR	1.59 (0.07)	1.69 (0.08)	1.59 (0.07)
p-value	0.392	0.737	0.361

Abbreviations: AL, ad libitum control group; CR, 25% calorie restriction group.

* Results are mean (SE). Predicted values based on statistical analysis.

† Significantly different from baseline within each treatment group at p<0.05.

‡ p<0.016 for response to *Tetanus toxoid*.

§ p<0.001 for total diameter of induration.

### Infection

Incidence of total infections or organ-specific infections, allergies and associated medications as well as severity of infections and allergies over the 24-month follow-up did not significantly differ between AL and CR groups (Supplemental Tables 3A and B). This was true for the annualized rate of infection and allergies with the exception of lower respiratory (CR group tended to have a lower rate 0.046 vs 0.015; p=0.058) and eye infections (CR group had a higher rate 0.00 vs 0.019; p=0.036) (Supplemental Table 3A).

## DISCUSSION

This is the first RCT to test the long-term effects of moderate CR without malnutrition in a large sample of young and middle-aged non-obese individuals using a variety of inflammatory and immune outcomes. We show that 25% CR for 24 mo persistently reduced circulating inflammatory markers including WBC count. Serum concentrations of CRP and TNF-α were about 40% and 50% lower in the CR group, respectively. Furthermore, despite a major reduction in body fat and circulating leptin levels, a significant impairment in key in vivo measures of adaptive immune function with CR was not observed in our study and this finding is supported by the lack of clinically significant differences in self-reported infection rate between CR and AL groups.

Low-grade chronic inflammation is implicated in the pathogenesis of multiple age-associated chronic diseases and in the biology of aging itself [4]. On the other hand, research on rodents housed in pathogen-free facilities and data from undernourished children and adults living in third world countries suggest that a chronic reduction in energy intake may impair adaptive immunity against pathogens by lowering leptin and other nutrient-sensing pathways [17, 20] While data from animal and observational human studies show that CR without malnutrition inhibits inflammation [3, 20-22], this RCT is the first to show a causal relationship in humans. The WBC count has been broadly used as a non-specific marker of systemic inflammation [23], with higher levels, even when within the clinical reference range, associated with an increased risk of developing insulin resistance, T2D [24], hypertension [25], CVD [26], and cancer [27]. Moreover, the relative risk of CVD and cancer mortality increases in a dose-dependent manner with increasing WBC count, independent of other risk factors [28]. Data from previous weight loss studies in obese individuals have shown that CR reduces total WBC count, IL-1β, IL-6, and TNF-α [29]. We found that CR induced a significant reduction in total WBC, lymphocyte and monocyte count, as well as a strong trend (p=0.067) for a decrease in neutrophils, suggesting that CR has metabolic benefits even in non-obese individuals. The anti-inflammatory effect of CR is further supported by the CR-induced decrease in serum levels of CRP, TNF-α, ICAM-1, and leptin [30]. However, in our study the serum concentrations of other pro-inflammatory cytokines and chemokines (IL-6, IL-8, MCP-1) were not significantly altered by CR, probably because our volunteers were healthy, young to middle-aged and non-obese, with relatively low levels of visceral adiposity [31]. Since obesity-associated increase in circulating IL-6 is mainly contributed by increased output from the visceral adipose tissue [31], it is possible that a reduction in visceral fat mass would lead to more pronounced IL-6-lowering effect in an obese individual relative to their non-obese counterpart with an already low IL-6 level.

The mechanisms underlying the anti-inflammatory effect of CR are not entirely clear. It is hypothesized that the reductions in fat mass and leptin largely explain the beneficial effect of CR on inflammation. However, our findings suggest that other metabolic and molecular factors may play a role, because peak reduction in circulating leptin levels at month 12 were not accompanied by a significant reduction in serum TNF-α levels. Thus, the significant reduction in CRP and TNF-α concentrations observed at 24 months may be due to CR-induced alterations of the neuroendocrine system through the down-regulation of nutrient-sensing pathways that impact mitochondrial function, redox status and inflammatory gene activation [32-35].

A major finding of this study is the lack of significant negative effects of CR on key *in vivo* indicators of cell-mediated immunity. There is controversy in the literature regarding the impact of CR on cell-mediated immunity. Although some animal studies indicate that age-associated impairment of immune function may be improved by CR, and short-term CR in a small number of subjects improved T cell-mediated function [36], others have raised concern regarding the potential adverse impact of CR on cell-mediated immunity and resistance to pathogens. For example, CR mice were shown to have lower natural killer cell activity, decreased survival, and delayed viral clearance compared to ad-libitum fed mice [13, 14], which can be reversed by re-feeding [37]. CR also caused higher mortality from polymicrobial sepsis [38] and West Nile Virus [39], and more susceptibility to the intestinal parasite (Heligmosomoides bakeri) infection [40] in mice. In this study, despite a ∼57% decrease in leptin, CR did not exert any detrimental effect on the two best available *in vivo* indicators of acquired (specific) immunity, i.e., antibody production to vaccines and DTH to recall antigens. This difference might be due to moderate level of CR (25%) administered in the current study compared to that used in several animal studies which can be as high as 40%. Taken together, these results suggest that moderate CR without malnutrition is safe and does not adversely affect immune response to pathogens, which is also supported by the lack of clinically significant differences in self-reported infection rate between CR and AL groups. It will be interesting to determine if lower than 25% CR would be effective in reducing inflammation.

In conclusion, data from this unique RCT showed that moderate long-term CR without malnutrition decreases inflammation in non-obese, healthy adults, as demonstrated by reduced number of WBC, lymphocytes, and neutrophils in blood, as well as reduced circulating levels of CRP, leptin, TNF-α, and ICAM-1, with no significant adverse effect on key *in vivo* indicators of cell-mediated immunity. These CR-induced changes suggest a shift toward a healthy phenotype, given the established role of these pro-inflammatory molecules as risk markers in the development of metabolic syndrome and age-related chronic diseases, in particular CVD, T2D and cancer.

## METHODS

### Overview

The Comprehensive Assessment of Long-term Effects of Reducing Intake of Energy (CALERIE) Phase 2 Study was a two-year, multi-center, parallel-group, single-blind RCT of healthy individuals receiving an intervention to reduce energy intake by 25% (CR) or maintain habitual ad libitum intake (AL-control) group. Clinical outcomes were assessed every 6-mo as detailed elsewhere [19, 41]. The study protocol (http://ClinicalTrials.gov ID:NCT00427193), was approved by the institutional review boards at all research sites, and participants provided written informed consent. Exclusion criteria for administration of vaccine and/or DTH included history of allergic reactions, infection or exposure to antibiotics in the previous two-weeks, non-steroidal anti-inflammatory drugs within 72 h, vaccination within last 6-wk, steroids >10 mg/d, or any immunosuppressive medication. For Hepatitis A only participants were screened out of the vaccination testing if they had previously received a vaccination.

Baseline testing was conducted over six weeks and included evaluations of health status and doubly labeled water (DLW) measurements of energy expenditure to individualize the 25% CR prescription. Fasting blood samples were collected for immune parameters. DTH and vaccines were administered as indicated below.

Following baseline testing, participants were randomized to either AL or CR in a 2:1 allocation in favor of CR. Randomization was stratified by site (3 sites), sex, and BMI (normal weight, overweight).

The intervention targeted an immediate and sustained 25% CR [42, 43]. Control participants were advised to continue their current diets. No specific level of physical activity was recommended. Percent CR was calculated and adherence evaluated from DLW measurements at months 12 and 24 [43]. Participants (both CR and AL) received a multivitamin and mineral supplement (Nature Made Multi Complete, Pharmavite, Mission Hills, CA) plus a calcium supplement (1000 mg/d, Douglas Laboratories, Pittsburgh, PA) to ensure current recommendations for micronutrients were met regardless of the intervention allocation.

### Outcome assessments

Participants were weighed in a pre-weighed hospital gown after an overnight fast (Scale Tronix 5200, White Plains, NY). Height was measured twice using a wall-mounted stadiometer. Percent body fat, lean mass, and bone were measured by dual X-ray absorptiometry (DXA; Hologic Inc., Bedford, MA) and analyzed using Hologic software version Apex 3.3.

As part of safety testing, participants record signs, symptoms, adverse events, and medication use in a diary and hematology, serum chemistry and urinalysis were monitored every 3-mo [41]. Self-reported infection, allergy, and antibiotic use and duration were recorded throughout the study and coded for severity (mild, moderate, and severe) by the Coordinating Center in accordance with MedRA version 14.1 and WHO Drug Dictionary Enhanced-March 2012 guidelines.

### Immune and inflammatory markers

Inflammatory markers were measured in fasting blood at baseline, month 12 and 24, and analyzed at the University of Vermont. Hs-CRP was measured using particle-enhanced immunonephelometric assay (BN II, Siemens, Deerfield, IL; CV=3.2 ± 2.5%); TNF-α, monocyte chemoattractant protein-1 (MCP-1), leptin, IL-1β, and IL-8 using the multiplex immunoassay (Human Adipokine Panel B, Millipore, Billerica, MA; Bio-Plex 200, Bio-Rad Laboratories, Hercules, CA; CV=6.1±1.7%, 6.4±2.1%, 4.3±1.5%, 8.0±4.4% and 8.7±3.9%, respectively); and IL-6 and intracellular adhesion molecule-1 (ICAM-1) using ELISA (R&D Systems, Minneapolis, MN; CV=7.9±3.1% and 8.2±1.2%, respectively). Complete blood count and WBC differential (CBC-Diff) were assayed using automated methods (Esoterix Inc., a LabCorp Company, Cranford, NJ).

*In vivo* cell-mediated immunity was assessed using delayed type hypersensitivity skin response (DTH) and antibody response to 3 vaccines. DTH, which determines ability of immune response to antigens to which it has been previously exposed, was assessed using Mantoux test. Three recall antigens [*Tetanus toxoid* (Aventis Pasteur), *Candida albicans* (Candin; Allermed Laboratories, San Diego, CA), and *Trichophyton* species (*Trichophyton mentagrophytes* in conjunction with *Trichophyton rubrum*; Hollister-Stier Labs, Spokane, WA)] and a negative control (0.9% normal saline) were used. Antigens were employed in a standard volume of 0.1 mL except *Tetanus toxoid* [0.025 mL (0.2 limit of flocculation units per dose)] and were injected intradermally on the volar surface of the forearm by trained research staff. Vertical and horizontal diameters of induration after 24 and 48 h were measured, and mean values >5 mm were considered positive. Total diameter of induration was calculated from sum of the means of the 3 antigens.

Antibody responses to vaccines were measured at the end of the intervention. Three vaccines, Hepatitis A (HEP-A) (primary T cell-dependent), tetanus/diphtheria (TD) (secondary T cell-dependent) and pneumococcal (B cell dependent) (PN) were administered at month 17. A booster shot for HEP-A was administered at month 23. Blood for antibody response was collected at month 17 (before vaccination), 18, and 24 (after vaccination) for all vaccines, and 23 for before HEP-A booster. Anti-HEP-A virus (anti-HAV) antibodies (total immuno-globulin, IgM and IgG) were measured by chemi-luminescent immunoassay (Elecsys, Roche Diagnostics, Indianapolis, IN; CV=1.9 ± 1.4%), anti-diphtheria, and anti-tetanus toxoid IgG antibodies by EIA, and anti-*Streptococcus pneumonia* IgG antibodies (23 serotypes) by microsphere photometry at Mayo Medical Laboratories, Rochester, MN.

### Complete blood count with differentials(CBC)

CBC and white cell differential were assayed using automated methods employd by Esoterix Inc. (A LabCorp Company, Cranford, NJ)

### Infection, asthma, allergies and antibiotic use

Self-reported infection, and asthma, allergy and antibiotic use, and their start and end date were recorded throughout the intervention period and coded based on severity (mild, moderate and severe) by the Coordinating Center in accordance with MedRA version 14.1 and WHO Drug Dictionary Enhanced-March 2012 guidelines.

### Statistical methods

Methods for the overall CALERIE study have been described elsewhere [19]. Briefly, intention-to-treat analysis was performed by including all available observations. For continuous outcomes (CBC-Diff and inflammatory markers) repeated Mixed models analysis [44-46] were used to examine change from baseline, controlling for site, sex, BMI stratum, and the baseline value for the outcome of interest. Significant between-group differences at each time point were tested at α=0.05. Bonferroni correction was applied where appropriate [47] for between group p values while within group changes p-values were always protected by a Bonferroni correction.

For values beyond the limits of detection of the assay for antibody response, a parametric regression model used in survival analysis [48] was applied. Values above or below detection limits were considered censored at those points. Between-group tests were performed using the lognormal distribution for the outcome adjusting for site, sex, and BMI stratum.

For the three DTH antigens, individual positive values were analyzed using the generalized estimating equation model [49] with the logit link and the Bernoulli variance. The number of positive antigens (0, 1, 2 or 3) was treated as a binomial outcome and analyzed in a similar manner. The induration diameters were treated as continuous and were analyzed using the repeated measures model described above.

The annualized infection, allergy, and associated medication rates were derived as the total number of episodes (or drugs) divided by the amount of follow-up time. For any outcome, a between-group comparison was performed using a generalized linear model [50] with the *ln* link and the Poisson variance, adjusting for site, sex and BMI stratum; the natural logarithm of the amount of follow-up time contributed by each participant was included as an offset. The incidence of any infection was treated as a binary outcome, and analyzed using the same *ln*-Poisson model with the modification suggested by Zou [51].

All analyses were performed by the statistical unit at Duke University Clinical Research Institute (DCRI, Durham, NC) using SAS software version 9.2 (SAS Institute Inc., Cary, NC).

## SUPPLEMENTAL DATA APPENDIX TABLES


