# Supplementary material for: Long-term moderate calorie restriction inhibits inflammation without impairing cell-mediated immunity: a randomized controlled trial in non-obese humans
Source: Aging (Albany NY). 2016 Jul 13;8(7):1416–26. doi: 10.18632/aging.100994 (PMC4993339; doi:10.18632/aging.100994)
Supplement: Supplementary file 1 [file aging-08-1416-s001.pdf]

## SUPPLEMENTAL DATA

**Appendix Table 1. Effect of calorie restriction on white blood cells***

| Variable (time) | Treatment Group | | p-value ‡ |
|---|---|---|---|
| | CR | AL | |
| Total monocytes ($10^3$/µL) | | | |
| Baseline | 0.33 (0.01) | 0.32 (0.01) | |
| Mo. 12 | 0.32 (0.01) | 0.37 (0.01) † | 0.004 |
| Mo. 24 | 0.32 (0.01) | 0.35 (0.01) † | 0.034 |
| Total Neutrophils ($10^3$/µL) | | | |
| Baseline | 3.68 (0.11) | 3.60 (0.13) | |
| Mo. 12 | 3.41 (0.12) † | 3.71 (0.16) | 0.106 |
| Mo. 24 | 3.24 (0.10) † | 3.54 (0.14) | 0.067 |
| Total Eosinophils ($10^3$/µL) | | | |
| Baseline | 0.14 (0.01) | 0.11 (0.01) | |
| Mo. 12 | 0.14 (0.01) | 0.12 (0.01) | 0.095 |
| Mo. 24 | 0.13 (0.01) | 0.15 (0.01) | 0.102 |
| Total Basophils ($10^3$/µL) | | | |
| Baseline | 0.01 (0.001) | 0.02 (0.002) | |
| Mo. 12 | 0.021 (0.002) † | 0.025 (0.002) † | 0.378 |
| Mo. 24 | 0.020 (0.002) † | 0.024 (0.002) † | 0.443 |

* Results are mean (SE) and reflect predicted values at months 12 and 24 based on intention-to-treat (ITT) statistical analysis, p-values reflect the ITT analyses for changes from baseline at each time point.

† Significantly different from baseline within the same treatment group.

‡ Values reflect difference in change from baseline between treatment groups at each time point.

| Appendix Table 2. Effect of calorie restriction on antibody response to Hepatitis A, Tetanus/Diphtheria and Pneumococcal vaccines | | | | | | |
|---|---|---|---|---|---|---|
| | Median (IQR)* | | | % with detectable values | | |
| Time (Mos.) | 17 | 18 | 24 | 17 | 18 | 24 |
| Hepatitis A (IU/L) | | | | | | |
| AL | 9.7 (7.8, 11.7) | 41.7 (29.5, 53.7) | NA | 42.9% | 27.5% | 0%† |
| CR | 8.6 (7.5, 10.3) | 37.3 (26.0, 53.0) | 28.5 (16.4, 40.7) | 28.0% | 23.2% | 5.1%† |
| p-value | 0.601‡ | 0.983‡ | NA | 0.670 | 0.832 | 0.332 |
| Tetanus (IU/mL) | | | | | | |
| AL | 2.43 (1.8, 3.5) | 4.95 (3.8, 5.9) | 3.31 (2.6, 4.1) | 90.2% | 51.7% | 79.3% |
| CR | 2.56 (1.7, 3.9) | 4.37 (3.3, 5.7) | 3.70 (2.6, 5.0) | 93.1% | 52.9% | 79.4% |
| p-value | 0.675 | 0.775 | 0.940 | 0.499‡ | 0.876‡ | 0.992‡ |
| Diphtheria (IU/mL) | | | | | | |
| AL | 0.36 (0.2, 0.7) | 1.1 (0.7, 1.9) | 0.77 (0.4, 1.1) | 100% | 93.3% | 98.3% |
| CR | 0.35 (0.2, 0.7) | 1.1 (0.7, 1.5) | 0.75 (0.4, 1.3) | 98% | 87.3% | 100% |
| p-value | 0.350 | 0.644 | 0.901 | 0.273 | 0.416 | 0.196 |
| Pneumonia IgG Serotype 1 (µg/mL) | | | | | | |
| AL | 1.5 (0.8, 4.5) | 10.0 (3.7, 35.4) | 8.1 (3.1, 30.5) | 85.7% | 95.1% | 100% |
| CR | 1.7 (0.8, 3.9) | 7.0 (2.5, 23.7) | 6.3 (2.9, 21.3) | 77.9% | 96.1% | 94.6% |
| p-value | 0.604 | 0.474 | 0.233 | 0.356 | 0.624 | 0.314 |
| Pneumonia IgG Serotype 2 (µg/mL) | | | | | | |
| AL | 0.65 (0.4, 1.3) | 5.5 (2.2, 9.3) | 4.3 (2.1, 12.5) | 88.9% | 94.3% | 100% |
| CR | 0.70 (0.4, 1.5) | 3.7 (1.7, 10.7) | 3.5 (1.4, 9.9) | 80.4% | 100% | 98.8% |
| p-value | 0.437 | 0.553 | 0.296 | 0.185 | 0.448 | 0.456 |
| Pneumonia IgG Serotype 3 (µg/mL) | | | | | | |
| AL | 1.0 (0.5, 2.8) | 3.8 (1.6, 8.0) | 2.9 (1.1, 7.7) | 90.5% | 98.4% | 98.1% |
| CR | 1.2 (0.5, 2.8) | 3.2 (1.8, 7.1) | 2.5 (1.2, 6.6) | 88.3% | 99.0% | 98.9% |
| p-value | 0.853 | 0.973 | 0.599 | 0.971 | 0.702 | 0.676 |
| Pneumonia IgG Serotype 4 (µg/mL) | | | | | | |
| AL | 0.5 (0.2, 1.6) | 1.3 (0.5, 3.6) | 1.1 (0.4, 3.9) | 76.2% | 100% | 100% |
| CR | 0.4 (0.2, 1.0) | 1.7 (0.9, 4.2) | 1.2 (0.7, 3.2) | 78.8% | 98.1% | 97.8% |
| p-value | 0.856 | 0.158 | 0.598 | 0.498 | 1.00 | 1.00 |
| Pneumonia IgG Serotype 5 (µg/mL) | | | | | | |
| AL | 2.8 (1.0, 5.2) | 4.7 (2.6, 13.4) | 4.1 (2.3, 11.3) | 96.8% | 100% | 100% |
| CR | 3.0 (1.2, 5.1) | 4.5 (2.0, 12.8) | 4.9 (2.1, 11.5) | 95.2% | 100% | 100% |
| p-value | 0.788 | 0.792 | 0.944 | 0.611 | 0.611 | 0.611 |
| Pneumonia IgG Serotype 8 (µg/mL) | | | | | | |
| AL | 1.1 (0.6, 2.6) | 3.5 (1.8, 7.5) | 3.0 (1.5, 6.9) | 100% | 100% | 100% |
| CR | 1.1 (0.5, 2.6) | 2.6 (1.2, 6.4) | 2.6 (1.3, 5.3) | 99.0% | 99.0% | 98.9% |
| p-value | 0.511 | 0.433 | 0.357 | 0.436 | 0.444 | 0.455 |
| Pneumonia IgG Serotype 14 (µg/mL) | | | | | | |
| AL | 3.2 (1.3, 5.2) | 11.7 (4.3, 30.6) | 13.6 (4.2, 29.4) | 74.6% | 93.4% | 86.5% |
| CR | 2.2 (1.2, 4.8) | 9.7 (2.8, 31.7) | 9.5 (3.0, 27.8) | 72.1% | 90.4% | 90.3% |
| p-value | 0.423 | 0.357 | 0.908 | 0.726 | 0.498 | 0.487 |
| Pneumonia IgG Serotype 20 (µg/mL) | | | | | | |
| AL | 0.8 (0.4, 1.8) | 3.0 (1.0, 11.9) | 2.5 (0.9, 5.3) | 79.4% | 98.4% | 96.2% |
| CR | 0.8 (0.5, 1.8) | 2.2 (0.9, 7.6) | 2.1 (0.7, 5.9) | 75.0% | 94.2% | 93.5% |
| p-value | 0.777 | 0.215 | 0.514 | 0.723 | 0.503 | 0.910 |
| Pneumonia IgG Serotype 6B (µg/mL) | | | | | | |
| AL | 2.5 (1.2, 5.2) | 5.1 (2.8, 11.9) | 4.3 (2.3, 10.2) | 85.7% | 93.4% | 92.3% |
| CR | 2.7 (1.4, 4.8) | 5.2 (3.0, 12.7) | 5.3 (2.8, 11.9) | 82.7% | 96.2% | 92.4% |
| p-value | 0.876 | 0.401 | 0.564 | 0.854 | 0.187 | 0.630 |

| Pneumonia IgG Serotype 7F (μg/mL) | | | | | | |
|---|---|---|---|---|---|---|
| AL | 3.0 (1.7, 6.8) | 5.2 (2.6, 11.4) | 4.8 (2.5, 9.6) | 98.4% | 96.7% | 98.1% |
| CR | 2.8 (1.5, 5.3) | 4.8 (2.3, 10.1) | 4.8 (2.7, 9.3) | 95.2% | 95.2% | 97.8% |
| p-value | 0.395 | 0.639 | 0.910 | 0.280 | 0.385 | 0.927 |
| Continued to next page | | | | | | |
| | Median (IQR)* | | | % with detectable values | | |
| Time (Mos.) | 17 | 18 | 24 | 17 | 18 | 24 |
| Pneumonia IgG Serotype 9N (μg/mL) | | | | | | |
| AL | 1.3 (0.7, 3.2) | 2.4 (1.2, 8.0) | 2.5 (1.2, 6.5) | 90.5% | 100% | 100% |
| CR | 1.4 (0.6, 3.5) | 2.8 (1.2, 6.0) | 2.7 (1.2, 6.3) | 77.9% | 95.1% | 95.7% |
| p-value | 0.140 | 0.633 | 0.674 | 0.083 | 0.734 | 0.457 |
| Pneumonia IgG Serotype 9V (μg/mL) | | | | | | |
| AL | 2.1 (1.0, 4.0) | 3.9 (1.3, 11.2) | 4.3 (1.7, 9.1) | 95.2% | 96.7% | 96.2% |
| CR | 1.9 (1.0, 4.7) | 3.4 (1.6, 8.2) | 3.5 (1.6, 8.4) | 92.3% | 96.2% | 96.8% |
| p-value | 0.636 | 0.162 | 0.215 | 0.461 | 0.851 | 0.845 |
| Pneumonia IgG Serotype 10A (μg/mL) | | | | | | |
| AL | 1.9 (0.9, 5.0) | 3.4 (1.6, 13.9) | 2.8 (1.3, 8.0) | 85.7% | 88.5% | 92.3% |
| CR | 2.7 (0.9, 5.0) | 3.8 (1.3, 9.5) | 3.0 (1.1, 8.0) | 76.9% | 89.4% | 89.2% |
| p-value | 0.393 | 0.681 | 0.325 | 0.168 | 0.859 | 0.551 |
| Pneumonia IgG Serotype 11A (μg/mL) | | | | | | |
| AL | 1.1 (0.5, 2.2) | 3.4 (1.9, 8.8) | 2.9 (1.5, 6.8) | 87.3% | 98.4% | 100% |
| CR | 1.2 (0.5, 4.2) | 4.1 (2.0, 8.6) | 3.6 (1.6, 8.1) | 94.2% | 100% | 98.9% |
| p-value | 0.093 | 0.799 | 0.905 | 0.118 | 0.194 | 0.455 |
| Pneumonia IgG Serotype 12F (μg/mL) | | | | | | |
| AL | 0.4 (0.2, 1.0) | 0.6 (0.3, 2.0) | 0.7 (0.3, 2.5) | 81.0% | 91.8% | 90.4% |
| CR | 0.6 (0.3, 2.1) | 1.2 (0.4, 3.9) | 1.0 (0.4, 4.1) | 74.0% | 92.3% | 89.2% |
| p-value | 0.626 | 0.211 | 0.395 | 0.308 | 0.908 | 0.830 |
| Pneumonia IgG Serotype 15B (μg/mL) | | | | | | |
| AL | 0.8 (0.4, 1.6) | 3.7 (1.7, 13.7) | 3.0 (1.5, 11.9) | 84.1% | 98.4% | 100% |
| CR | 0.8 (0.4, 2.0) | 4.1 (1.6, 13.0) | 4.0 (1.6, 10.4) | 84.6% | 98.1% | 96.8% |
| p-value | 0.577 | 0.650 | 0.672 | 0.933 | 0.452 | 0.192 |
| Pneumonia IgG Serotype 17F (μg/mL) | | | | | | |
| AL | 3.2 (1.5, 6.4) | 12.1 (4.7, 21.9) | 10.3 (4.0, 19.8) | 85.7% | 98.4% | 100% |
| CR | 3.0 (1.6, 6.0) | 11.1 (5.0, 24.8) | 10.8 (5.7, 19.4) | 89.4% | 98.1% | 97.8% |
| p-value | 0.739 | 0.785 | 0.897 | 0.476 | 0.896 | 0.289 |
| Pneumonia IgG Serotype 18C (μg/mL) | | | | | | |
| AL | 0.6 (0.3, 1.4) | 3.5 (1.0, 10.2) | 3.4 (1.1, 9.4) | 68.3% | 98.4% | 92.3% |
| CR | 0.8 (0.5, 2.0) | 4.4 (1.5, 10.8) | 4.3 (1.3, 8.6) | 71.2% | 98.1% | 97.8% |
| p-value | 0.239 | 0.303 | 0.344 | 0.693 | 0.896 | 0.109 |
| Pneumonia IgG Serotype 19A (μg/mL) | | | | | | |
| AL | 4.7 (2.6, 12.5) | 8.5 (3.6, 24.8) | 11.3 (4.8, 23.7) | 76.2% | 88.3% | 86.3% |
| CR | 4.3 (2.3, 9.4) | 13.0 (5.7, 38.0) | 12.9 (4.8, 39.2) | 79.4% | 89.0% | 89.9% |
| p-value | 0.798 | 0.451 | 0.581 | 0.628 | 0.611 | 0.957 |
| Pneumonia IgG Serotype 19F (μg/mL) | | | | | | |
| AL | 2.7 (1.3, 5.1) | 5.2 (2.6, 16.6) | 3.9 (2.0, 13.8) | 74.6% | 86.9% | 90.4% |
| CR | 2.9 (1.5, 5.6) | 8.4 (2.9, 18.0) | 5.9 (2.5, 12.0) | 84.6% | 91.3% | 93.5% |
| p-value | 0.063 | 0.228 | 0.256 | 0.062 | 0.200 | 0.254 |
| Pneumonia IgG Serotype 22F (μg/mL) | | | | | | |
| AL | 4.2 (2.1, 8.0) | 5.2 (2.7, 10.2) | 4.9 (2.5, 10.6) | 96.8% | 95.1% | 98.1% |
| CR | 3.3 (1.6, 8.8) | 3.6 (2.0, 10.1) | 4.5 (2.0, 9.0) | 91.3% | 96.2% | 95.7% |
| p-value | 0.165 | 0.413 | 0.379 | 0.031 | 0.503 | 0.453 |

| | | | | | | |
|---|---|---|---|---|---|---|
| Pneumonia IgG Serotype 23F (μg/mL) | | | | | | |
| AL | 7.8 (3.2, 16.4) | 8.8 (3.3, 20.0) | 8.1 (3.4, 21.0) | 95.2% | 93.4% | 96.2% |
| CR | 7.5 (2.8, 16.2) | 9.3 (3.7, 21.0) | 9.6 (3.5, 18.8) | 92.3% | 94.2% | 93.5% |
| p-value | 0.532 | 0.726 | 0.505 | 0.461 | 0.524 | 0.511 |
| Pneumonia IgG Serotype 33F (μg/mL) | | | | | | |
| AL | 1.2 (0.6, 2.9) | 4.3 (1.3, 13.0) | 4.4 (1.5, 11.2) | 85.7% | 100% | 98.1% |
| CR | 0.9 (0.5, 2.4) | 5.0 (1.8, 9.6) | 4.2 (1.8, 12.5) | 89.4% | 96.2% | 97.8% |
| p-value | 0.363 | 0.636 | 0.980 | 0.285 | 0.444 | 0.441 |

* Based on observations within detectable range. IQR, Inter-Quartile Range
† For Hepatitis A values reflect those above the detectable range.
‡ p-values are from statistical analysis accounting for censoring.
NA: Since majority of values were beyond the detectable range quantitative analysis could not be performed.

**Appendix Table 3A. Annualized Rate of Infections, Allergies and Associated Medications Over the 24-Month Follow-up**

| | **AL (N=75)** | | | **CR (N=143)** | | | |
|---|---|---|---|---|---|---|---|
| **Event** | **Total No. Episodes*** | **Average No. Days†** | **Annualized Rate‡** | **Total No. Episodes*** | **Average No. Days†** | **Annualized Rate‡** | **p-value§** |
| **All Infections** | 156 | 14.5 | 1.032 | 239 | 9.8 | 0.897 | 0.2838 |
| Total respiratory infections | 115 | 11.9 | 0.761 | 169 | 7.2 | 0.635 | 0.2099 |
| Upper respiratory infections | 108 | 10.9 | 0.715 | 165 | 6.9 | 0.620 | 0.3684 |
| Lower respiratory infections | 7 | 1.0 | 0.046 | 4 | 0.2 | 0.015 | 0.0583 |
| GI infections | 7 | 0.2 | 0.046 | 18 | 0.4 | 0.068 | 0.3602 |
| Skin infections | 9 | 0.7 | 0.060 | 7 | 0.2 | 0.026 | 0.1161 |
| Urinary track infections | 6 | 0.5 | 0.040 | 11 | 0.4 | 0.041 | 0.9154 |
| Ear infections | 4 | 0.3 | 0.026 | 2 | 0.1 | 0.008 | 0.1420 |
| Eye infections | 0 | 0.0 | 0.000 | 5 | 0.1 | 0.019 | 0.0357 |
| Oral dental infections | 6 | 0.3 | 0.040 | 19 | 0.9 | 0.071 | 0.1445 |
| **Allergies** | 36 | 1.3 | 0.238 | 47 | 1.2 | 0.176 | 0.1912 |
| **OTC Medication Use** | 190 | 145.0 | 1.257 | 346 | 229.9 | 1.299 | 0.7120 |
| **Allergy Medication Use** | 82 | 55.5 | 0.543 | 138 | 50.8 | 0.518 | 0.7871 |
| **Antibiotics Medication Use** | 71 | 35.6 | 0.470 | 139 | 33.9 | 0.522 | 0.4516 |

* Total number of distinct episodes summed across participants in that treatment arm.
† The total number of days during which the event was prevalent across participants in that treatment arm, divided by the number of participants in that treatment group
‡ Total number of distinct events divided by the total amount of follow-up time in that treatment arm, standardized to 365 days in a calendar year.
§ The p-value is derived from the Poisson regression model comparing the number of distinct episodes between the two groups.

| **Appendix Table 3B. Distribution of the Severity of the Events, Pooled Across all Events Among Participants Who Experienced the Event at Least Once** | | | | | | | | |
|---|---|---|---|---|---|---|---|---|
| | **AL (N=75)** | | | | **CR (N=143)** | | | |
| **Event** | **No. Pts >=1 event*** | **Mild†** | **Moderate** | **Severe** | **No. Pts >=1 event*** | **Mild†** | **Moderate** | **Severe** |
| **All Infections** | 53 | 70 (44.9%) | 64 (41.0%) | 22 (14.1%) | 89 | 99 (41.4%) | 111 (46.4%) | 29 (12.1%) |
| Total respiratory infections | 48 | 51 (44.3%) | 51 (44.3%) | 13 (11.3%) | 79 | 71 (42.0%) | 77 (45.6%) | 21 (12.4%) |
| Upper respiratory infections | 46 | 49 (45.4%) | 47 (43.5%) | 12 (11.1%) | 79 | 70 (42.4%) | 74 (44.8%) | 21 (12.7%) |
| Lower respiratory infections | 5 | 2 (28.6%) | 4 (57.1%) | 1 (14.3%) | 4 | 1 (25.0%) | 3 (75.0%) | 0 |
| GI infections | 7 | 2 (28.6%) | 1 (14.3%) | 4 (57.1%) | 14 | 6 (33.3%) | 8 (44.4%) | 4 (22.2%) |
| Skin infections | 7 | 6 (66.7%) | 3 (33.3%) | 0 | 7 | 3 (42.9%) | 3 (42.9%) | 1 (14.3%) |
| Urinary track infections | 4 | 2 (33.3%) | 3 (50.0%) | 1 (16.7%) | 8 | 1 (9.1%) | 10 (90.9%) | 0 |
| Ear infections | 3 | 0 | 1 (25.0%) | 3 (75.0%) | 2 | 0 | 0 | 2 (100.0%) |
| Eye infections | 0 | 0 | 0 | 0 | 4 | 3 (60.0%) | 2 (40.0%) | 0 |
| Oral dental infections | 4 | 3 (50.0%) | 3 (50.0%) | 0 | 7 | 10 (52.6%) | 8 (42.1%) | 1 (5.3%) |
| **Allergies** | 16 | 27 (75.0%) | 6 (16.7%) | 3 (8.3%) | 23 | 28 (59.6%) | 13 (27.7%) | 6 (12.8%) |

* Number of participants who experienced the event at least once in that treatment arm.
† The frequency and percent of all such events pooled across all events across all participants who experienced that event at least once.
